# Society Notes

**Published:** 1903-08

**Authors:** E. O. Boggs


### Society Notes.

*Dr. F. E. Daniel, Austin, Texas.*

DEAR DOCTOR: Dr. Sam R. Burroughs, councilor for the Eleventh District, organized the Leon County Medical Association at this place on the 22nd instant, with a charter membership of 19. The following officers were elected: Dr. S. R. Burroughs, Buffalo, President; Dr. W. T. Evans, Jewett, Vice-President; Dr. J. H. Joyce, Buffalo, Secretary-Treasurer. Dr. E. O. Boggs, Marquez, Dr. D. W. Montgomery, Centerville, Dr. H. H. Thompson, Leona, compose the board of censors, and Dr. Z. J. Spruiell, of Jewett, was elected delegate to the State Association. Old Leon will be in the front rank.

Yours fraternally,

E. O. BOGGS.

